# Developing Cathode Films for Practical All‐Solid‐State Lithium‐Sulfur Batteries

**DOI:** 10.1002/adma.202407738

**Published:** 2024-07-29

**Authors:** Chao Ye, Shijie Xu, Huan Li, Jieqiong Shan, Shi‐Zhang Qiao

**Affiliations:** ^1^ School of Chemical Engineering The University of Adelaide Adelaide SA 5005 Australia; ^2^ Department of Chemistry City University of Hong Kong Kowloon Hong Kong 999077 P. R. China

**Keywords:** all‐solid‐state batteries, lithium‐sulfur batteries, sulfur‐based cathode films

## Abstract

The development of all‐solid‐state lithium‐sulfur batteries (ASSLSBs) toward large‐scale electrochemical energy storage is driven by the higher specific energies and lower cost in comparison with the state‐of‐the‐art Li‐ion batteries. Yet, insufficient mechanistic understanding and quantitative parameters of the key components in sulfur‐based cathode hinders the advancement of the ASSLSB technologies. This review offers a comprehensive analysis of electrode parameters, including specific capacity, voltage, S mass loading and S content toward establishing the specific energy (Wh kg^−1^) and energy density (Wh L^−1^) of the ASSLSBs. Additionally, this work critically evaluates the progress in enhancing lithium ion and electron percolation and mitigating electrochemical‐mechanical degradation in sulfur‐based cathodes. Last, a critical outlook on potential future research directions is provided to guide the rational design of high‐performance sulfur‐based cathodes toward practical ASSLSBs.

## Graphical abstract

The development of all‐solid‐state lithium‐sulfur batteries offers higher specific energies and lower costs compared to state‐of‐the‐art Li‐ion batteries. However, a lack of mechanistic understanding hinders advancement. This review analyzes key electrode parameters, evaluates progress in enhancing ion/electron percolation, and addresses electrochemical‐mechanical degradation, offering future research directions.

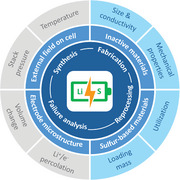

## 1 Introduction

Developing batteries with high specific energy is crucial for advancing energy storage technologies, particularly for practical applications including the electrification of vehicles, marine shipping, and aviation. According to the United States Advanced Battery Consortium (USABC), cell‐level batteries should achieve specific energies and costs of over 1000 Wh kg^−1^ and $ 0.3 kWh^−1^ for energy storage systems and aviation, achieving these benchmarks is challenging.^[^ ^1^ ^]^ One of the most promising strategies to achieve high specific energy is constructing all‐solid‐state lithium metal batteries (ASSLMBs) by replacing the widely used graphite anode (372 mAh g^−1^) with Li metal anode (3860 mAh g^−1^), with the safety concerns addressed by using non‐flammable solid‐state electrolytes (SEs). So far, tremendous efforts have been devoted to fabricating SE films with high ionic conductivity, high chemical/electrochemical stability and intimate SE/electrode interface.

The lithium‐sulfur batteries (LSBs) characterized by the S redox reaction S_8_ + 16Li ↔ 8Li_2_S offers a high theoretical capacity of 1675 mAh g^−1^ and voltage near 2.2 V relative to Li^+^/Li.^[^ ^2^ ^]^ Consequently, they provide a significantly higher theoretical specific energy and energy density compared to conventional lithium‐ion batteries, reaching up to 2,500 Wh kg^−1^ and 2,800 Wh L^−1^, respectively.^[^ ^3^ ^]^ In addition, the abundant availability of elemental sulfur contribute to the relative low estimated cost. These factors position all‐solid‐state lithium‐sulfur batteries (ASSLSBs) as a highly attractive candidate among all‐solid‐state lithium metal battery systems.^[^ ^4^, ^5^ ^]^

As the critical component, the active sulfur‐based materials in the cathode films determine the capacity and specific energy of the ASSLSBs. Therefore, achieving a high loading and content of sulfur‐based active materials in the cathode film is of paramount importance. Additionally, factors such as discharge voltage and specific capacity are essential for achieving high specific energy. However, developing sulfur‐based cathodes with SEs faces significant challenges compared to that with non‐aqueous electrolytes. These challenges include Li^+^ conductive behaviors between the SE and sulfur‐based materials particles, kinetically sluggish redox reactions of solid sulfur redox reaction.^[^ ^6^, ^7^ ^]^ More importantly, electrochemical‐mechanical degradation originated from significant volume change during cycling is more profound in solid‐state sulfur‐based cathode film.^[^ ^8^ ^]^ Therefore, a timely review is needed for assessing the critical parameters and guiding the rational design of high‐specific‐energy solid‐state sulfur‐based cathode films.

Herein, we start from systematic review on recent progress in the practical ASSLSBs at pouch‐cell level and analysis quantitative parameters of the specific energy and energy density. In addition, we investigate active materials synthesis, reprocessing and cathode film fabrication. In addition, we overview the design of practical solid‐state sulfur‐based cathode films with Li^+^ and electron percolation. Subsequently, the strategies to suppress the electrochemical‐mechanical coupling effect are reviewed. Finally, an outlook on the future directions regarding development of sulfur‐based cathode films is provided to offer a promising roadmap toward higher‐specific‐energy ASSLSBs.

## 2 Evaluation of Specific Energy and Energy Density of the ASSLSBs

With the development of laboratory‐scale fundamental research, all‐solid‐state lithium‐sulfur pouch batteries have been studied. While facing challenges of air stability, electrochemical stability, and compatibility with positive/negative electrodes, sulfide SEs with high ionic conductivity and deformability are recognized as one of the most promising SEs.^[^ ^9^ ^]^

### 2.1 Electrochemical Properties of the ASSLSBs at Pouch‐Cell Level

Recent studies have demonstrated the pouch‐cell ASSLSBs, for example, ASSLSB with a reversible capacity of 1067 mAh g^−1^ in 30 cycles under sulfur loading of 4.5 mg cm^−2^ and current of 1 mA cm^−2^ (**Table** **1**).^[^ ^10^ ^]^ Another report fabricated sheet‐type sulfur cathodes by a dry process, in which the binder was 0.1 wt% with a thickness of 50 to 300 µm. This sulfur cathode achieved longer than 50 cycles with high capacity retention under sulfur loading of 1.5 mg cm^−2^.^[^ ^11^ ^]^ Other reports also demonstrated successful fabrication of ASSLSBs at pouch‐cell level.^[^ ^12^, ^13^, ^14^ ^]^ Compared to all‐solid‐state pouch cells based on metal oxides active materials, all‐solid‐state sulfur‐based pouch cells generally have lower active material content and specific areal loading. Both types of all‐solid‐state pouch cells find it challenging to achieve higher current densities, with most cases being below 2 mA cm^−2^.^[^ ^15^ ^]^ This indicates that in scaling up pouch cell experiments, fast electron and ion transport under high current conditions remains a significant challenge in solid‐state pouch cells.^[^ ^16^ ^]^ In addition, the cycling performance of all‐solid‐stat pouch cells based on metal oxides active materials is significantly better than that of all‐solid‐state sulfur‐based pouch cells.^[^ ^17^ ^]^ This also implies that the sulfur‐based cathode films encounters more severe issues during cycling, such as volumetric expansion and solid electrolyte decomposition.

**Table 1:** Representative all‐solid‐state pouch cells with sulfur‐based cathodes and transitional metal oxide‐based cathodes.

Cathode	Content [%]	Materials loading [mg cm^−2^]	Solid‐state electrolyte	Electrolyte thickness [µm]	Anode	Temperature [°C]	Stack pressure [MPa/kPa]	Current density [mA cm^−2^]	Voltage [V]	Initial capacity [mAh g^−1^]	Capacity retention [%]@cycle	Refs.^a^ ^)^
AB^b)^@S	40	4.5	LiSiPSCl^n)^ @PTFE	200	Li_3.75_Si	60	270 MPa	1.0	1–2.6	1512	70.6@30	[10]
CB^c)^@S	30	1.8	LPSCl@ PTFE	900	Li‐In	30	500 MPa	0.15	1–2.4	1500	99@30	[11]
C/S/LGPS‐SR^m)^	25	0.5	LPSCl	380	Li	60	300 MPa	0.01	1.5–2.8	1169	81@10	[12]
S@C @LPS^f)^	25	2	LPSCl@ PTFE	−	Li	60	−	0.17	1.5 –2.8	767	−	[13]
LPSCl^g)^ @CNT^e)^@Si@S	30	2.5	LPSCl@Si rubber@CEL^p)^	60	Li‐In	25	5–10 kPa	0.21	0.8–2.4	1064	−	[14]
Li_3_InCl_6_@LiCoO_2_‐PTFE^h)^	−	−	Li_3_InCl_6_‐LPSCl	20	Graphite‐LPSCl‐PTFE	25	50 MPa	−	1.8–4.2	121.2	68.6@50	[119]
NMC^i)^ @LZO^j)^	85	≈30	LPSCl‐xylene	30–40	Ag‐Carbon	60	490 MPa	3.40	2.5–4.25	146	89@1000	[17]
NCM^i)^ @LNO^k)^	85	−	LPSCl‐PTFE	150	Graphite‐LPSCl‐PTFE	25	300 MPa	0.70	2.5–4	184	93.6@100	[120]
Mo_6_S_8_	70	3.9	LGPS^l)^‐CY52‐005^o)^	10–20	Li‐Al	25	100 MPa	0.64	0.5–3.5	82	48@8	[121]
Li_4_Ti_5_O_12_	49.8	15	LPS‐Ni@ PPTA^d)^‐LPS	70	LiCoO_2_	30	370 MPa	0.11	1–3	80	−	[122]

^a)^ The data are collected or derived from literature without considering the current collector;

^b)^ AB: Acetylene Black;

^c)^ CB: Carbon Black;

^d)^ PPTA: Poly(paraphenylene terephthalamide);

^e)^ CNT: Carbon Nanotube;

^f)^ LPS: Lithium Phosphorus Sulfide;

^g)^ LPSCl: Lithium Phosphorus Sulfide Chloride;

^h)^ PTFE: Polytetrafluoroethylene;

^i)^ NCM or NMC: Nickel Cobalt Manganese ternary cathode;

^j)^ LZO: Lithium Zirconium Oxide;

^k)^ LNO: Lithium Nickel Oxide;

^l)^ LGPS: Lithium Germanium Phosphorus Sulfide;

^m)^ SR: Silicone Rubber;

^n)^ LiSiPSCl: Lithium Silicon Phosphorus Sulfide Chloride;

^o)^ CY52‐005: Cross‐linkable Polysiloxane;

^p)^ CEL: Cellulose Fiber.

### 2.2 Specific Energy and Energy Density of the ASSLSBs and Quantitative Parameters

Among various cell types, pouch cell with light package materials shows higher specific energy, thus is suitable for developing ASSLSBs. The reported size and areas of the pouch cell model used in this calculation for ASSLSBs include the areas of sulfur‐based cathode (A_cathode_ = 87.6 cm^2^), SE (A_SE_ = 95.8 cm^2^) and Li metal electrode (A_Li_ = 93.5 cm^2^).^[^ ^9^ ^]^

The ASSLSB is composed of S‐based cathode with sulfur, SE (with fixed mass ratio of 15% in the cathode), electronically conductive additive and binder (with fixed mass ratio of 0.2% in the cathode). Important parameters are therefore, molar mass of S (M_s_), areal mass loading of S (m_sulfur_), mass ratio of S in the sulfur‐based electrode (R_cathode_), molar mass of the Li metal electrode (M_Li_), negative/positive electrode capacity ratio (R_N/P_), areal mass of the solid electrolyte (m_SE_, based on the density ρ_SE_ of 1.9 g mL^−1^ and the thickness T_SE_), areal mass of the Li metal electrode (m_Li_, based on the R_N/P_), areal mass of Al and Cu current collector (m_Al_ and m_Cu_, based on the thickness of T_Al_ and T_Cu_), mass of the package in the “whole” cell (m_package_, 6.4 g). The specific capacity of the S electrode can be determined as C_sulfur_. In addition, the mean average discharge cell voltage can be determined as V_mean_, therefore, the specific energy (Wh kg^−1^) of the pouch cell is calculated from:

(1) $$\begin{array}{ccc} & & \mathrm{Specific}\;\mathrm{energy}\;\left(\mathrm{Wh}\;kg^{-1}\right)\\ & = & \frac{A_{\mathrm{cathode}}\;\times \;C_{\mathrm{sulfur}}\;\times \;V_{\mathrm{mean}}\;\times \;m_{\mathrm{sulfur}}}{A_{\mathrm{cathode}}\;\times \;\frac{m_{\mathrm{sulfur}}}{R_{\mathrm{cathode}}}+m_{\mathrm{Al}}+m_{\mathrm{Cu}}+m_{\mathrm{package}}+A_{\mathrm{SE}}\;\times \;\rho_{\mathrm{SE}}\;\times \;T_{\mathrm{SE}}+A_{\mathrm{Li}}\;\times \;\frac{V_{s}\;\times \;M_{\mathrm{Li}}}{V_{\mathrm{Li}}\;\times \;M_{s}}\;\times \;R_{N/P}\;\times \;m_{\mathrm{sulfur}}} \end{array}$$

The relationship between the specific capacity and voltage, S loading and S content of the cathodes can therefore be established.^[^ ^18^ ^]^ In addition, the densities of sulfur, carbon and binder are represented by ρ_sulfur_, ρ_carbon_ and ρ_binder_. The porosity of the sulfur electrode is set as 0.25. In addition, based on measured thickness and volume of a practical pouch cell including T_package_ (152 µm), T_0_ (7788 µm) and V_0_ (0.09 L),^[^ ^9^ ^]^ the energy density (Wh L^−1^) can be calculated following the equation:

(2) $$\begin{array}{ccc} & & \mathrm{Energy}\;\mathrm{density}\left(\mathrm{Wh}\;L^{-1}\right)\\ & = & \frac{A_{\mathrm{cathode}}\;\times \;C_{\mathrm{sulfur}}\;\times \;V_{\mathrm{mean}}\;\times \;m_{\mathrm{sulfur}}\;\times \;T_{0}}{\left(\frac{m_{\mathrm{sulfur}}/\rho_{\mathrm{sulfur}}+m_{\mathrm{carbon}}/\rho_{\mathrm{carbon}}+m_{\mathrm{SE}}/\rho_{\mathrm{SE}}+m_{\mathrm{binder}}/\rho_{\mathrm{binder}}}{1-V_{\mathrm{pore}}}+T_{\mathrm{Al}}+T_{\mathrm{Cu}}+T_{\mathrm{package}}+T_{\mathrm{SE}}+\frac{m_{\mathrm{Li}}}{\rho_{\mathrm{Li}}}\right)\;\times \;V_{0}} \end{array}$$

where ρ_sulfur_, ρ_carbon_ and ρ_binder_ represent the densities of sulfur, carbon and binder, and the porosity of the sulfur electrode is set as 0.25.

**Figure 1:**
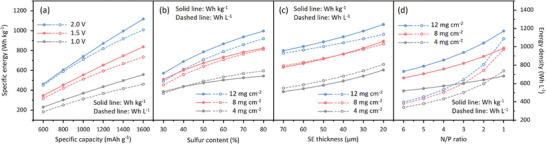
Specific energies, energy densities and related quantitative parameters of the ASSLSBs at pouch‐cell level. a) Calculated specific energy and energy density with specific capacity and average discharge cell voltage. b) Calculated specific energy and energy density with S content and S loading mass in the cathode. c) Calculated specific energy and energy density with SE thickness and S loading mass. d) Calculated specific energy and energy density with negative‐positive electrode capacity ratio (R_N/P_) and S loading.

Accordingly, the impact of a series of key parameters on the specific energy and energy density of ASSLSBs can be discussed. As shown in **Figure** **1**a, increasing specific capacity of cathode results in a nearly linear increase in specific energy and energy density of the ASSLSBs. For example, when the specific capacity increases from 600 to 1600 mAh g^−1^ with an average discharge cell voltage of 2.0 V, the pouch cell's specific energy and energy density are boosted from 464 Wh kg^−1^ and 582 Wh L^−1^ to 1118 Wh kg^−1^ and 1185 Wh L^−1^, respectively. Similarly, the average discharge cell voltage V_mean_ significantly affects both specific energy and energy density of ASSLSB pouch cells via a near‐linear relationship. In addition, as illustrated in Figure 1b, increasing S loading m_sulfur_ from 4 to 12 mg cm^−2^ results in a significant boost to specific energy and energy density from 544 Wh kg^−1^ and 738 Wh L^−1^ to 997 Wh kg^−1^ and 1088 Wh L^−1^ of ASSLSB pouch cells with 80% S content. It is also demonstrated that when the S content is >70%, its impact on boosting specific energy is limited. Therefore, specific capacity, discharge voltage, S loading, and S content are the key parameters in sulfur‐based cathode that determine the specific energy and energy density of the ASSLSB pouch cells.

Apart from the parameters of sulfur‐based cathode, those of the other components also significantly impact the specific energy and energy density of the ASSLSB pouch cells. For example, the thickness of solid electrolyte T_SE_ demonstrates a negative correlation with the specific energy and energy density (Figure 1c). Notably, when the S loading is larger than 8 mg cm^−2^, its impact on boosting pouch‐cell specific energy and energy density is limited. Therefore, T_SE_ is a major restraining parameter for specific energy and energy density of the high‐sulfur‐loading ASSLSBs. For example, under the S loading of 8 mg cm^−2^, decreasing T_SE_ from 70 to 20 µm results in significant boost in specific energy and energy density from 525 Wh kg^−1^ and 381 Wh L^−1^ to 825 Wh kg^−1^ and 973 Wh L^−1^, respectively. Practically, 30 µm can be regarded as suitable T_SE_ of solid electrolyte thin film. Additionally, the impact of R_N/P_ on pouch‐cell specific energy and energy density was determined against that of S loading, Figure 1d. For example, if a ASSLSB pouch cell with a S loading of 4 mg cm^−2^ and R_N/P_ of 6 exhibits specific energy and energy density of 395 Wh kg^−1^ and 339 Wh L^−1^, which can be significantly increased respectively to 544 Wh kg^−1^ and 738 Wh L^−1^ when decreasing R_N/P_ to 1, whilst increasing S loading to 12 mg cm^−2^ results in increased values of 590 Wh kg^−1^ and 397 Wh L^−1^. This finding evidence that R_N/P_ is an important parameter for developing high‐energy‐density ASSLSBs.

With the development of all‐solid‐state batteries, the strategies for suppressing lithium dendrites, stabilizing anode and cathode interface, and improving conductivity of solid electrolytes developed for ASSLMBs can be leveraged to enhance the interface stability and overall electrochemical performance of ASSLSBs.^[^ ^19^ ^]^ Moreover, advanced manufacturing techniques from metal oxide‐based cathodes in ASSLMBs can be applied to sulfur‐based cathodes in ASSLSBs to reduce electrode thickness and increase energy density.Among various determining parameters in ASSLSB, achieving a low N/P ratio (approaching 1) and exploring thin (less than 30 µm), highly conductive solid electrolyte membranes that are stable with lithium metal are crucial. More importantly, developing thick solid‐state sulfur‐based cathodes with an aerial capacity exceeding 16 mAh cm^−2^, a cathode sulfur loading exceeding 12 mg cm^2^ and a sulfur utilization of 90% is of high importance to realize high‐energy ASSLSBs with >1000 Wh kg^−1^ (**Table** **2**).

**Table 2:** Parameters of the projected all‐solid‐state Li|NMC811 and ASSLSBs pouch cell.

Cell component	Cell parameters	All‐solid‐state Li||NMC811 Pouch Cell	ASSLSB Pouch Cell	High‐energy ASSLSB Pouch Cell
Cathode	first discharge capacity [mAh g^−1^]	200	1200	1400
	Active material content [%]	80	50	80
	Total coating weight [mg cm^−2^ each side]	26	10	15
	Areal capacity [mAh cm^−2^ each side]	4.2	6.0	16.8
	Electrode press density [g cm^−3^]	3.4	1.5	1.5
	Electrode length [cm]	12	12	12
	Electrode width [cm]	7.3	7.3	7.3
	Thickness [single side, µm]	77	67	98
	Al foil thickness [µm]	16	16	16
	Layers	22	22	22
Li anode	N/P ratio	2	2	1
	Thickness [single side, µm]	40.7	58.7	82.1
	Cu foil thickness (µm)	8	8	8
Solid electrolyte	Thickness (µm)	30	30	30
Packaging foil	Thickness (µm)	152	152	152
Cell	voltage (V)	308	2	2
	Capacity (Ah)	16.0	23.1	64.8
	Specific energy [Wh kg^−1^]	373	438	997
	Energy density [Wh L^−1^]	636	505	1088

## 3 Synthesis and Reprocessing of the Sulfur‐Based Active Materials

The synthesis and reprocessing methods for the active sulfur‐based materials is the basic part to determining battery performance.^[^ ^20^ ^]^ While elemental sulfur is currently the mainstream active material for ASSLSBs, its inherently low electronic and ionic conductivity and severe volumetric expansion and contraction remain challenging to address.^[^ ^21^ ^]^ Researchers have explored various inorganic sulfides: lithium sulfide and transition metal sulfides.^[^ ^22^, ^23^ ^]^ As the final discharge product of elemental sulfur, lithium sulfide can also serve as an active material, delivering a specific capacity of 1166 mAh g^−1^. The density of lithium sulfide (1.66 g cm^−3^) is lower than that of elemental sulfur (2.07 g cm^−3^), leading to volume contraction during charging and expansion during discharging. This mitigates structural damage to the cathode and represent a significant advantage compared with elemental sulfur.^[^ ^24^ ^]^ In addition, transition metal sulfides such as CoS,^[^ ^25^ ^]^ TiS_2,_^[^ ^26^ ^]^ Mo_2_S,^[^ ^27^ ^]^ FeS_2_,^[^ ^28^ ^]^ and NiS^[^ ^29^ ^]^ have gained research interest. Although these transition metal sulfides sacrifice a part of theoretical specific capacity, the incorporation of transition metals significantly enhances overall conductivity compared to elemental sulfur and lithium sulfide, making these materials promising in ASSLSBs.

### 3.1 Synthetic Methods of the Sulfur‐Based Active Materials

**Figure 2:**
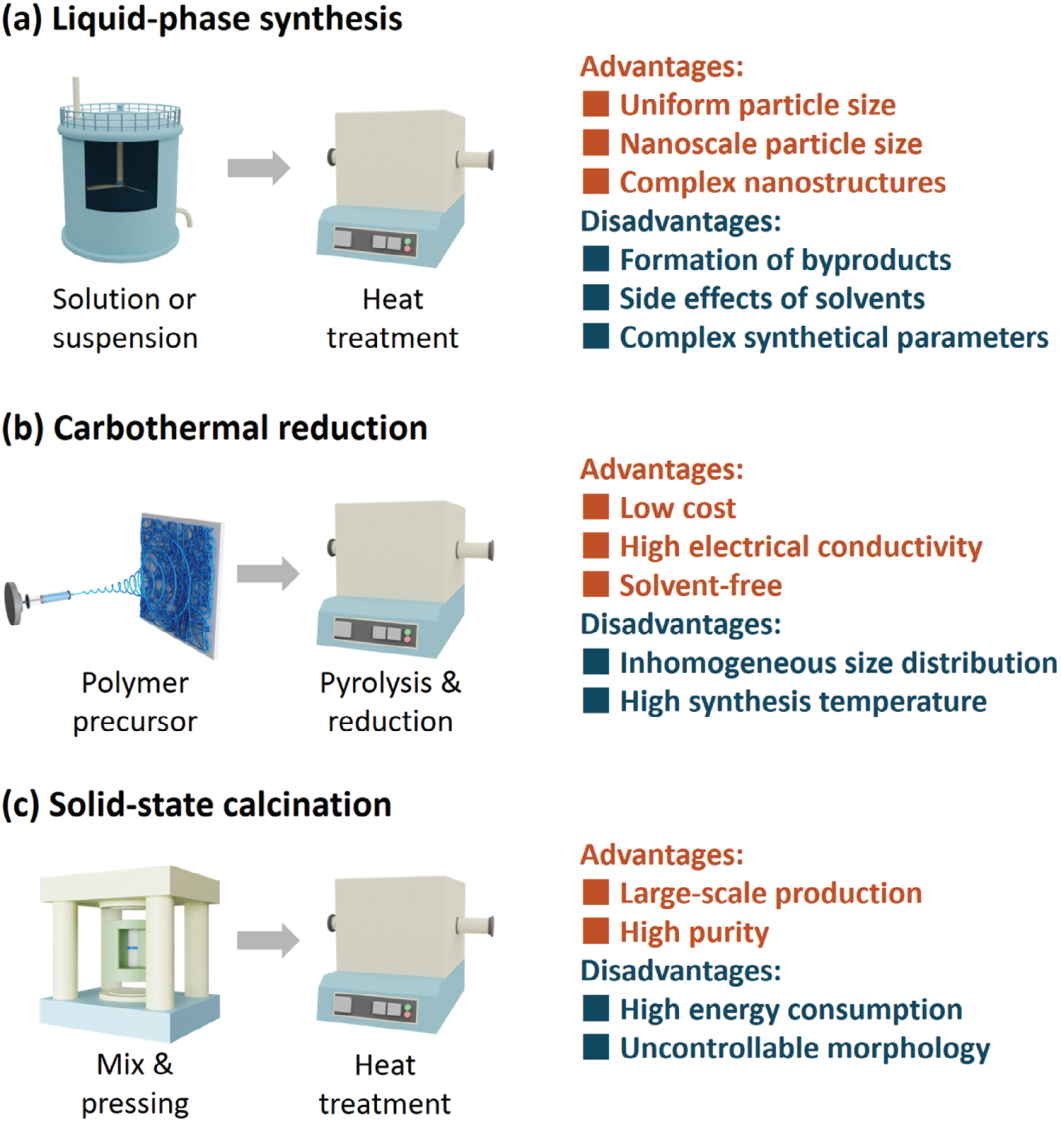
Synthetic methods of the active sulfur‐based materials with advantages and disadvantages. Schematics of a) liquid‐phase synthesis, b) carbothermal reduction, and c, solid‐state calcination.

The advantages of liquid synthesis lie in its relatively mild reaction conditions, avoiding the need for high temperatures and pressures, thus reducing energy consumption costs and equipment requirements (**Figure** **2**a). For example, Zheng et al. synthesized sulfur nanoparticles by reacting sodium thiosulfate (Na_2_S_2_O_3_) with dilute hydrochloric acid (HCl) under the action of the surfactant Polyvinyl Pyrrolidone (PVP), and loaded them onto lithium lanthanum titanium oxide/carbon nanofibers (LLTO/C) with mixed ionic/electronic conductivity. The sulfur prepared via the liquid‐phase method achieved about 9 nm nanoparticle size and formed a uniform composite structure with the nanofibers. Compared to the three‐phase interface formed through ball milling, this nano‐scale composite enables efficient charge transfer at the interface without disrupting the microstructure of the materials, thereby enhancing the solid‐state electrochemical reactions of sulfur. Consequently, this all‐solid‐state sulfur cathode exhibits high sulfur utilization (69%) and excellent rate performance (500 mAh g^−1^ at 1.0 C).^[^ ^30^ ^]^

Compared to the elemental sulfur, the liquid‐phase synthesis of lithium sulfide offers more opportunities.^[^ ^31^ ^]^ Nazar et al. synthesized nanoscale lithium sulfide particles by reducing elemental sulfur with lithium foil in a lithium naphthalenide solution, and further reacted it with VCl_4_ in tetrahydrofuran to prepare Li_2_S‐LiVS_2_ core‐shell structures. The core‐shell morphology achieved close contact between the quasi‐metallic LiVS_2_ shell and the insulating Li_2_S core, and the two‐phase boundaries providing well‐connected electronic and ionic pathways around the active material, enabling the battery to be charged at relatively high current densities up to 3 mA cm^−2^.^[^ ^32^ ^]^

Yao et al. utilized a multi‐step wet chemical liquid‐phase synthesis to form sulfur‐rich MoS_6_‐based nanocomposites (MoS_6_‐CNT20). The (NH_4_)_2_Mo_2_S_12_·2H_2_O precursor reacts with iodine in a DMF solvent containing carbon nanotubes. Additionally, Li_7_P_3_S_11_ electrolyte was coated onto the surface of MoS_6_‐CNT20 to construct a nanoscale electronic/ionic transport network. The resulting sulfur‐rich MoS_6_‐based nanocomposites cathode exhibited high electronic and ionic conductivity, with an initial discharge capacity of 1034 mAh g^−1^ at 0.1 A g^−1^. Furthermore, the ultra‐high reversible energy density of the active material reached 1640 Wh kg^−1^, the highest among all transition metal sulfide cathodes. Although liquid‐phase synthesis is beneficial for designing and achieving complex nanostructures, it is challenging to control the morphology and particle size of different nanomaterials within the same liquid phase, thus requiring continuous exploration and precise regulation of synthetical parameters.^[^ ^33^ ^]^ In addition, the use of large quantities of organic solvents and precursors in liquid‐phase synthesis not only increases the risk of environmental pollution but also requires the product to undergo a series of purification and washing processes.

The carbothermal reduction method for producing lithium sulfide has been extensively developed and applied in the industrial production of soda since the 19th century (Figure 2b).^[^ ^34^ ^]^ Under the protection of inert gas, both elemental carbon and organic carbon chains can serve as reducing agents to reduce lithium sulfate (Li_2_SO_4_) at high temperatures, producing Li_2_S and CO_2_.^[^ ^35^, ^36^ ^]^ Compared to liquid‐phase synthesis, the carbothermal reduction method does not require expensive organic solvents and precursors. Carbon and lithium sulfate as raw materials can be applied for simple and efficient large‐scale production of lithium sulfide. However, on an industrial scale, the carbothermal reduction method produces microcrystals Li_2_S with particle sizes ranging from 50 to 100 µm.^[^ ^37^ ^]^ Because the particle size of lithium sulfide prepared by this method depends not only on the lithium sulfate raw material but also on the continuous growth of Li_2_S crystals during the high‐temperature reduction process.^[^ ^38^ ^]^

Currently, at the laboratory scale, some studies have integrated processes such as ball milling, spin coating, freeze‐drying, and spray‐drying to obtain smaller particle sizes of Li₂S.^[^ ^39^ ^]^ Elemental carbon, due to its abundance, is widely used as a reducing agent for synthesizing Li_2_S‐C composites. Xie et al. prepared Li_2_S‐acetylene black (AB) composites by thoroughly mixing AB and lithium sulfate (Li_2_SO_4_) in a specific mass ratio, then calcining and reducing the mixture at 800 °C under an argon atmosphere for 5 h. This method heats lithium sulfate to approach its melting point for reduction, resulting in lithium sulfide that retains the morphology of the lithium sulfate but with smaller particle sizes. This increases the utilization of lithium sulfide active material in the sulfur cathode (90% at 0.2 mA cm^−2^), accelerating electrode reaction kinetics, and improving the cycling performance and rate capability of the ASSLSBs. Specifically, this Li_2_S‐based all‐solid‐state sulfur cathode with a loading of 5.78 mg cm^−2^ achieves an aerial capacity of 5.52 mAh cm^−2^ at a current density of 0.8 mA cm^−2^.^[^ ^40^, ^41^ ^]^

In addition to the unique carbothermal reduction method, solid‐state calcination under high temperatures is also an efficient solvent‐free synthetical method to prepare lithium sulfide and transition metal sulfides (Figure 2c). This method involves driving chemical reactions between solid reactants through high temperatures. Currently, industrial production of Li_2_S through high‐temperature solid‐state calcination can achieve relative low‐cost, large‐scale production. Compared to the expensive organic solvents and precursors used in liquid‐phase synthesis, raw materials such as lithium sulfate (Li_2_SO_4_), lithium carbonate (Li_2_CO_3_), lithium hydroxide (LiOH), and hydrogen sulfide (H_2_S) have abundant reserves.^[^ ^34^ ^]^ Jacob et al. reported a high‐temperature solid‐state calcination method that can achieve Li_2_S with a purity exceeding 98%. Initially, Li_2_CO_3_ powder, with a particle size distribution of 1–10 µm, is thoroughly contacted with H_2_S at a temperature of 150–175 °C, forming a protective Li_2_S coating on the surface of Li_2_CO_3_. Then, the mixture is further heated to ≈500–700 °C in an H_2_S atmosphere. Due to the melting point 938 °C of Li_2_S, this protective Li_2_S coating can prevent the Li_2_CO_3_ particles from sintering during calcination, resulting in a final Li_2_S product with smaller particle size and more uniform distribution.^[^ ^34^ ^]^ Adelhelm et al. synthesized highly crystalline CuS active materials with excellent conductivity by high‐temperature solid‐state calcination of ball‐milled sulfur and copper powder mixtures. The CuS active material prepared by this method exhibits an electrical conductivity of 870 S cm^−1^, eliminating the dependence on carbon conductive agents.^[^ ^42^ ^]^

### 3.2 Reprocessing of the Sulfur‐Based Active Materials

Although sulfur‐based electrode materials synthesized through industrial production are low‐cost, they are generally difficult to directly employ in ASSLSBs. The particle size, particle size distribution, conductivity, and purity of the active materials significantly impact the electrochemical activity in the sulfur cathode. Therefore, reprocessing techniques are widely used to improve the electrochemical properties of commercially purchased elemental sulfur, lithium sulfide, and transition metal sulfides, including ball milling, melt diffusion and vapor deposition, and dissolution‐recrystallization processes.^[^ ^43^ ^]^

**Figure 3:**
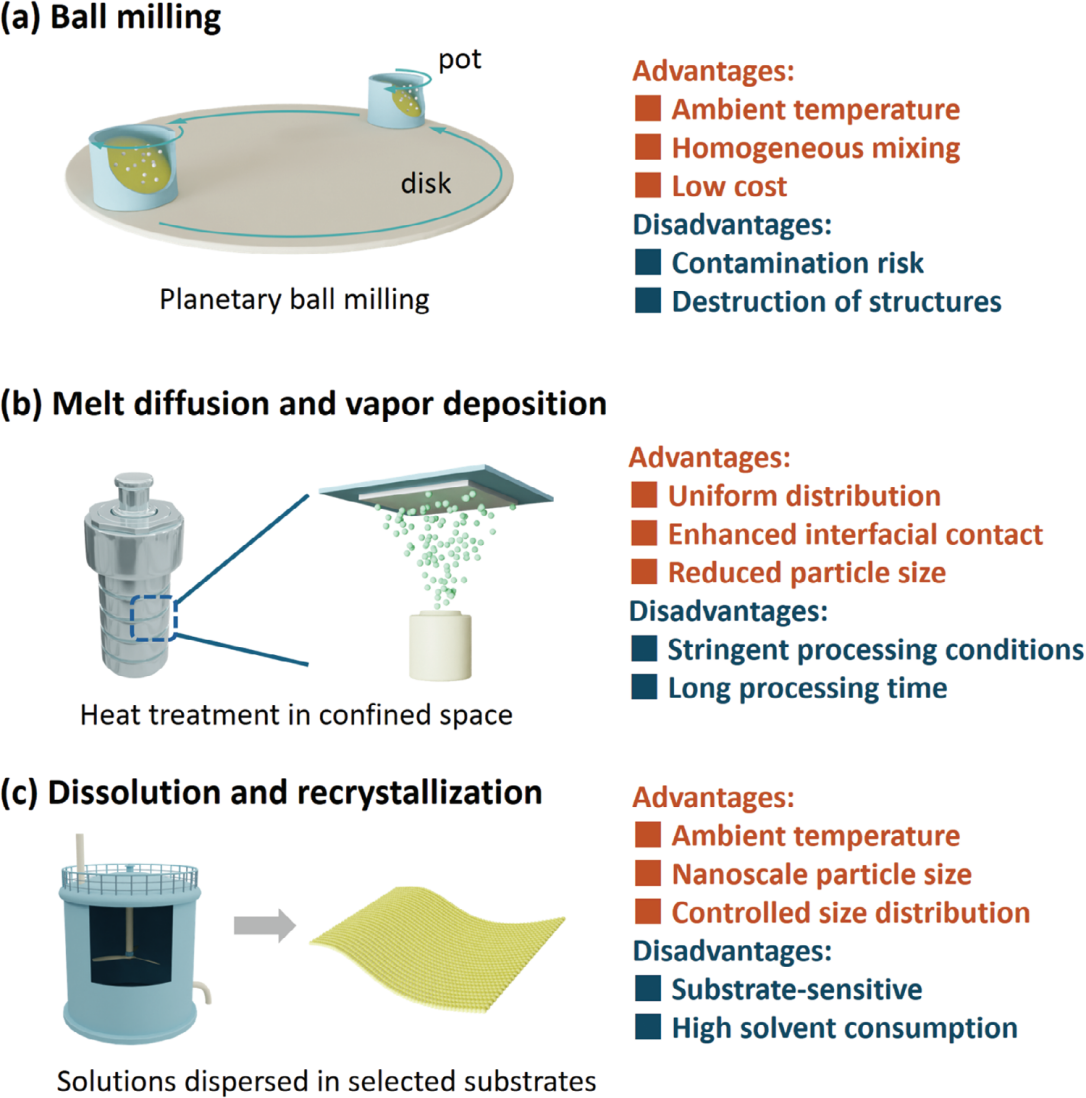
Reprocessing approaches of commercial sulfur‐based materials with advantages and disadvantages. Schematics of a) ball milling process, b) melt diffusion and vapor deposition, and c) dissolution and recrystallization process.

Ball milling is extensively used for comminuting and grinding large particles into smaller particles (**Figure** **3**a).^[^ ^44^ ^]^ In ASSLSBs, the active material, solid electrolyte, and conductive agent are thoroughly and uniformly mixed via ball milling, constructing continuous electronic and ionic microstructures. These mixed electronic‐ionic pathways significantly impact the electrochemical performance.^[^ ^45^ ^]^ Zeier et al. prepared sulfur cathodes using two different mixing methods: manual grinding and ball milling. The results showed that the composite sulfur cathode prepared by manual grinding had an initial capacity of only 220 mAh g^−1^ and suffered nearly half capacity loss during the subsequent charging process. In contrast, the composite sulfur cathode material prepared by ball milling delivered over 1000 mAh g^−1^ and exhibited minimal capacity loss during the charging process. By achieving smaller particle sizes of the active material through ball milling, the structural damage to the electrode caused by volume changes can be minimized, thereby enabling higher capacity and excellent cycling stability.^[^ ^46^ ^]^ By ball milling under an argon atmosphere, the particle size of Li_2_S can even be controlled to below 100 nm. Shaw et al. attempted to form a composite system by thoroughly milling carbon black and commercially available Li_2_S powders, followed by mixing the composite with pyrrole and calcining it at high temperatures, resulting in nitrogen‐doped composite structures after pyrrole pyrolysis.^[^ ^47^, ^48^ ^]^ In large‐scale applications, ball milling suffers from issues such as long process duration and contamination risk.^[^ ^49^ ^]^ In addition, ball milling is not suitable for processing certain complex nanostructures, such as nanotubes and nanoparticles, which are prone to be damaged during the milling process.^[^ ^50^ ^]^

Elemental sulfur has prompted the development of melt diffusion and vapor deposition techniques in sulfur cathodes due to its low melting point and ease of sublimation. (Figure 3b).^[^ ^51^, ^52^, ^53^ ^]^ Typically, only porous nanomaterials with high specific surface area, high porosity, and appropriate pore structure can produce a capillary effect that effectively anchors and adsorbs sulfur.^[^ ^54^ ^]^ Sun et al. mixed elemental sulfur with self‐assembled flower‐like MoS_2_ porous nanomaterials and heated the mixture to 155 °C. This promotes close contact between the active material and the conductive support. The ASSLSBs achieved a specific capacity of 1487.58 mAh g^−1^ at 0.1 C.^[^ ^55^ ^]^ However, the flow of liquid sulfur in melt diffusion is a slow process that requires a long processing duration. Additionally, it is difficult to ensure a uniform sulfur coating thickness on the support surface, often resulting in large sulfur clusters.^[^ ^56^ ^]^ Compared to other techniques, melt diffusion and vapor deposition require operations in an inert gas‐filled sealed environment under high temperatures. These processes are time‐consuming, and the stringent operating conditions may limit their industrial scalability. Furthermore, the high processing temperatures make these methods not suitable for composite systems containing polymers or those sensitive to high temperatures.^[^ ^57^ ^]^

The dissolution and recrystallization process are a common method for purifying commercially purchased or synthesized S and Li_2_S powders. (Figure 3c). S and Li_2_S powders can dissolve in various organic solvents, including ethanol, carbon disulfide, and ethylenediamine. After sufficient dissolution, the solvent is evaporated to recrystallize the materials, generating nanoscale active particles.^[^ ^38^ ^]^ This method allows for the simple and efficient transformation of active materials into nanoscale particles and their uniform loading onto corresponding substrates.^[^ ^58^ ^]^ During the recrystallization process, controlling the nucleation and growth of crystals is key to managing particle size. The method involves directly mixing the dissolved solution of active materials with dispersions of other nanomaterials, leveraging the surface polarity of the substrate materials to promote nucleation and growth of nanoscale particles.^[^ ^59^ ^]^ Yao et al. successfully prepared composite of carbon nanotubes and sulfur by mixing a solution of sulfur dissolved in ethylenediamine with a dispersion of carbon nanotubes. The all‐solid‐state lithium‐sulfur battery exhibited a capacity of 660.3 mAh g^−1^ after 400 cycles at a high rate of 1 C.^[^ ^60^ ^]^ Another method involves adding surfactants to the dissolved solution. Wu et al. used polyvinylpyrrolidone (PVP) as a surfactant to form a homogeneous solution with Li_2_S and ethanol. During the evaporation of ethanol, Li_2_S nanoparticles nucleated and precipitated. Due to the strong affinity between the polar functional groups of PVP and Li_2_S, PVP wrapped around the nanoparticles during nucleation and growth, preventing excessive growth of the nanoparticles. SEM images showed that these Li_2_S nanoparticles were controlled within the size range of 100–400 nm. The sulfur cathode prepared under this strategy retained a discharge capacity of 1200 mAh g^−1^ after 100 cycles.^[^ ^61^ ^]^ The extensive use of organic solvents in this method can result in high costs and environmental pollution issues. Additionally, this approach requires stringent synthesis conditions, solution purity,and precise control of solution temperature and cooling rates to improve reproducibility and stability.

## 4 Preparation Processes of the Sulfur‐Based Cathode Film

Achieving an aerial capacity > 6 mAh cm^−2^ is crucial for ASSLSBs to attain an energy density exceeding 500 Wh kg^−1^ (Table 2).^[^ ^62^ ^]^ Therefore, the preparation of the cathode films has a decisive impact on the final electrochemical performance of the ASSLSBs.^[^ ^63^ ^]^ Poor electrode‐electrolyte interface contact, significant interfacial side reactions, and inefficient charge transfer kinetics due to high tortuosity in the paths for lithium ion and electron transport within thick electrodes are major factors leading to the degradation of performance in all‐solid‐state lithium‐sulfur pouch batteries.^[^ ^64^ ^]^ In this chapter, we focus on the promising manufacturing processes for thick sulfur cathodes, including slurry casting, dry film technology, and in situ polymerization. We also employ the Technology Readiness Level (TRL) to evaluate the development stages of the technology, in which TRL 1–4 represent the research laboratory stage at the levels of coin cell (mAh), and small prototype (mAh‐Ah). TRL 5–6 denote the stage of pilot plant, particularly focusing on ampere‐hour scale‐up of pouch cells. TRL 7–10 denotes the commercial stage.^[^ ^65^ ^]^

### 4.1 Wet Process

**Figure 4:**
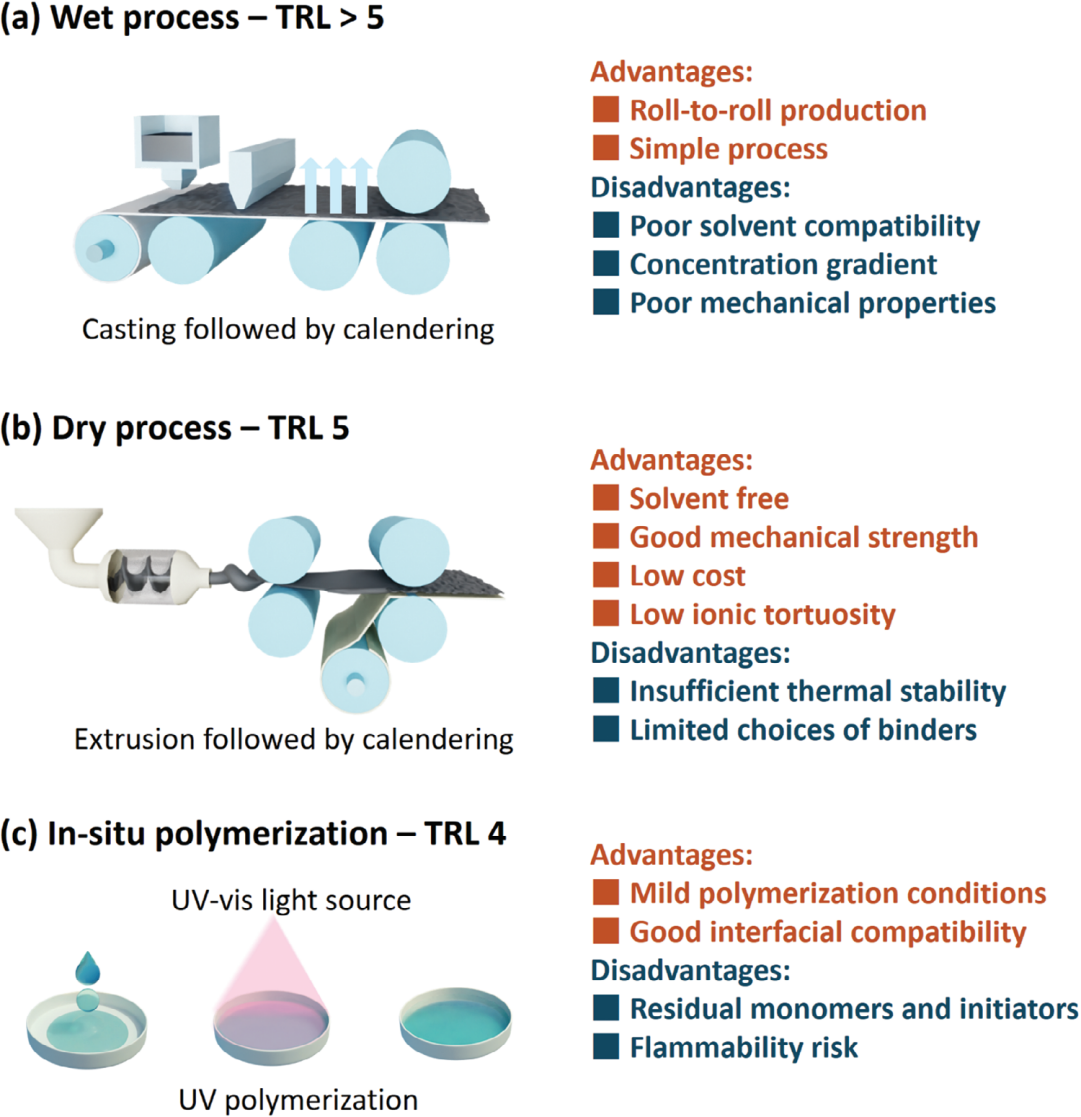
Preparation of the sulfur‐based cathode films with TRLs, advantages and disadvantages. Schematics of a) slurry casting followed by calendaring process; b) extrusion followed by sintering or calendaring process; and c) in situ polymerization process.

The slurry casting process is a simple, efficient, and scalable wet processing technique, which is widely used for preparing various electrode material films.^[^ ^66^ ^]^ The interface compatibility and mechanical properties of the prepared electrode films grant the slurry casting a TRL > 5 for roll‐to‐roll scaling potential (**Figure** **4**a).^[^ ^12^, ^67^, ^68^ ^]^ Zhang et al. successfully prepared sheet‐type sulfur cathode film by selecting weakly polar, low dielectric constant n‐hexane solvent and optimizing the content of silicone rubber (SR) binder in the slurry casting process, achieving a discharge capacity of 2.3 mAh cm^−2^.^[^ ^12^ ^]^ This indicates that solvent selection and binder content have significant effects on the microstructure of the entire electrode in the slurry casting process. In the preparation of sulfur cathodes for ASSLSBs, commonly used sulfide or halide solid‐state electrolytes are sensitive to many organic solvents. The poor compatibility results in a series of deactivation phenomena, including dissolution, complexation, and degradation of the solid‐state electrolytes. Therefore, the selection and matching of appropriate solid‐state electrolytes, solvents, and binders become core challenges of this process. In the practical slurry casting process, the extensive use of toxic and expensive organic solvents not only leads to residual solvents in the film but also results in environmental pollution and high costs. Even with prolonged drying, it is difficult to completely remove small molecule solvents from the product.^[^ ^69^, ^70^ ^]^ The slurry casting process for thick electrodes faces specific challenges, such as sedimentation and the formation of concentration gradients during prolonged drying. This inhomogeneous distribution of active materials and binders results in poor mechanical strength.^[^ ^71^, ^72^, ^73^ ^]^

### 4.2 Dry Process

The dry process, an emerging solvent‐free processing technology, has garnered significant attention from academia and industry in recent years.^[^ ^74^, ^75^ ^]^ Compared to the slurry casting process, this dry film technology inherently avoids the porosity and cracking issues caused by solvent evaporation, thereby enhancing the ion transport and mechanical strength of the electrode films (Figure 4b). Maxwell Technology claims that their dry process technology has achieved a production speed comparable to traditional slurry coating processes (25 m min^−1^) during pilot‐scale production. This evidently indicates that the TRL of this technology has reached level 5.^[^ ^74^, ^76^, ^77^ ^]^ The dry process can reduce the electrode porosity to 4%−10%, a substantial decrease compared to the 56% porosity when using slurry casting.^[^ ^72^, ^78^ ^]^ Kaskel et al. explored the impact of binder content in the dry process on the electrochemical performance and mechanical strength of sulfur cathodes in ASSLSBs. They achieved the lowest reported polytetrafluoroethylene (PTFE) binder content for dry film sulfur cathodes (as low as 0.1 wt%). This reduced PTFE content allows the binder to maintain the film's mechanical strength without interfering with the ionic and electronic pathways, resulting in good rate capability and cycling performance.^[^ ^11^ ^]^ Selecting suitable binders and optimizing process parameters are crucial for the commercial scalability of dry process technology. Researchers have also attempted to develop alternative binders, such as styrene‐butadiene rubber (SBR) and silicone rubber (SR), but their viscosity and plasticity remain inferior to PTFE.^[^ ^79^ ^]^

### 4.3 In Situ Polymerization Process

In situ polymerization, developed in recent years for the preparation of polymer‐based solid‐state batteries, is an advanced, simple, and efficient processing technique. It can be categorized into two main types: in situ thermal polymerization and in situ UV polymerization (Figure 4c).^[^ ^80^ ^]^ This establishes good interfacial compatibility, wettability, and stable interfacial contact between the electrode and the polymer. Moreover, compared to the traditional solution casting method for polymer‐based electrolytes, the in situ polymerization process significantly reduces the extensive use and evaporation of solvents, thereby minimizing solvent waste and effectively lowering production costs. Shi et al. employed azobisisobutyronitrile (AIBN) as an initiator, where the radicals generated from the decomposition of AIBN effectively attacked polyethylene glycol diacrylate (PEGDA) monomers. This process triggered an in situ crosslinking polymerization reaction within the sulfurized polyacrylonitrile‐based (SPAN‐based) cathode, forming a network‐structured polymer‐based solid electrolyte. The PEGDA‐based polymer solid electrolyte exhibited a high ionic conductivity of 6.87 × 10^−3^ S cm^−1^ at 30 °C. The Li|PEGDA|SPAN battery maintained a discharge specific capacity of 1217.3 mAh g^−1^ after 250 cycles at 0.2 C.^[^ ^81^ ^]^ In addition, the in situ polymerization strategy helps to construct continuous Li⁺ transport pathways, reducing the interfacial impedance between the electrode and electrolyte.^[^ ^82^ ^]^ This is crucial for maintaining the integrity of ion transport paths in thick sulfur cathodes and extending the lifespan of solid‐state lithium‐sulfur batteries. However, the polymer system still faces the challenge of polysulfide shuttle effects.^[^ ^83^, ^84^ ^]^ The in situ polymer electrolytes still face challenges of residual monomers, free radical and oligomers.^[^ ^85^ ^]^ Currently, the TRL of in situ polymerization remains at 4, limited to experimental validation at the small prototype scale.^[^ ^83^, ^86^, ^87^ ^]^

## 5 Strategies to Develop Sulfur‐Based Cathodes Film for Practical ASSLSBs

Due to the insulative property of the sulfur species, the solid redox reactions necessitate an effective combination of sulfur with conductive agents and solid‐state electrolytes to facilitate the concurrent transfer of electrons and lithium ions.^[^ ^88^ ^]^ The efficient and uniform construction of the tri‐phase interface comprising sulfur, conductive agents, and solid‐state electrolytes within the sulfur cathode through refined processes and strategies is a primary consideration for the future commercialization of the ASSLSBs.

### 5.1 Li Ion and Electron Percolation in the Sulfur‐Based Cathodes

In the case of sulfur‐based cathode films, due to the insulating character of sulfur, both ionic and electronic percolation are critical to fully utilize the theoretical capacity.^[^ ^89^ ^]^

This can be intrinsically improved by doping sulfur species to enhance the electronic and ionic percolation. For example, an S_9.3_I molecular crystal with I_2_ inserted into the crystalline sulfur structure has been recently reported, which exhibits electrical conductivity of ≈5.9 × 10^−7^ S cm^−1^ at 25 °C, which is an 11‐order‐of‐magnitude increase over the pristine sulfur.^[^ ^90^ ^]^ Additionally, the introduction of selenium (Se) into sulfur cathodes by forming SeS_x_ solid solutions have been reported to modify electronic and ionic conductivities, ultimately enhancing sulfur utilization. This originates from the redistribution of electron densities upon introducing Se demonstrated by theoretical calculations. The ionic conductivities of the achieved SeS_x_‐Li_3_PS_4_ (x = 3, 2, 1, and 0.33) composites is 10^−6^ S cm^−1^. the SeS_2_/Li_10_GeP_2_S_12_‐Li_3_PS_4_/Li solid‐state cells exhibit a high capacity of over 1100 mAh g^−1^ at current density of 50 mA g^−1^.^[^ ^91^ ^]^ The neutron imaging characterization demonstrated that sluggish Li ion transport within the composite cathode is the rate‐limiting process. The tomography reveals irreversibly concentrated lithium in the vicinity of the current collector, highlighting a sluggish Li ion transport within the sulfur‐based cathode.^[^ ^92^ ^]^ The adoption of electrolyte with high ionic conductivity has also been investigated to enhance the Li^+^ percolation of the ASSLSBs. For example, Li_6_PS_5_Br with ultrafast room temperature ionic conductivity of 2.58 × 10^−3^ mS cm^−1^ serves as an attractive candidate electrolyte. Neutron diffraction and X‐ray diffraction analyses show that the high ionic conductivity is originated from the high purity, short lithium ion jumps, and optimal Br ordering. As a result, the ASSLSBs using the optimized Li_6_PS_5_Br electrolyte demonstrate higher capacities.^[^ ^93^ ^]^

In addition, constructing a sulfur‐based cathode with nanosized sulfur‐based materials, solid electrolyte, and carbon can be a promising approach to mitigate the challenges regarding electronic and ionic percolation. For example, a nanocomposite was synthesized with Li_2_S, polyvinylpyrrolidone as the carbon precursor, and Li_6_PS_5_Cl as the solid electrolyte. This homogeneous nanocomposite serves as a mechanically robust and mixed conductive sulfur electrode. The obtained ASSLSBs with a high Li_2_S loading of ≈3.6 mg cm^−2^ demonstrate a capacity of 830 mAh g^−1^ under 50 mA g^−1^.^[^ ^94^ ^]^

**Figure 5:**
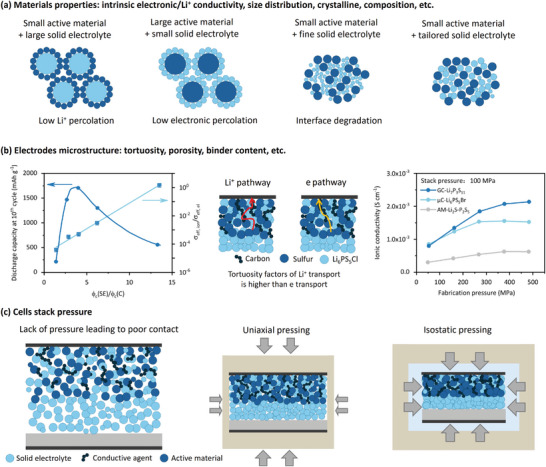
Strategies to improve Li ion and electron percolation in thick sulfur‐based cathode films. a) From the level of the materials, the effect of size distribution of the active materials and solid electrolyte. Reproduced with permission.^[^ ^95^ ^]^ 2022, John Wiley and Sons. b) From the level of the electrodes, microstructure including tortuosity, porosity, fabrication pressure. Reproduced with the permission.^[^ ^89^, ^97^ ^]^ 2021, John Wiley and Sons. 2021, American Chemical Society. c) From the level of the cell, application of stack pressure has been proved effective.

Several factors should be considered when designing cathode active materials and their particle size distributions, as summarized in **Figure** **5**a.^[^ ^89^ ^]^ Key parameters such as transport pathways and active interface area are influenced by the particles size distribution of both active materials and solid electrolyte. While smaller particles offer advantages in terms of percolation and shorter diffusion pathways, their high specific surface area can pose additional challenges. Therefore, tailored particles size distribution of active materials and solid electrolyte are necessary.^[^ ^95^ ^]^ As both employed sulfur and carbon are small particles, the SE morphology, particle sizes and size distribution affect the tortuosity of solid‐state cathodes. For crystalline thiophosphate electrolyte, the temperature‐assisted ball‐milling or solution‐based synthesis in combination with short annealing may represent promising strategies for retaining its high ionic conductivity when mixing with sulfur and carbon species.

The left panel in the Figure 5b illustrates the specific discharge capacity of the 10th cycle and the ratio of effective ionic to effective electronic conductivity as a function of the volume ratio of solid electrolyte to carbon for ASSLSBs, identifying ionic conduction as a bottleneck. The optimal discharge capacity is observed at a volume ratio of ≈4.^[^ ^89^ ^]^ From the electrode level, there are a few critical factors, including tortuosity, porosity, and binder content of electrode that impact the electronic and ionic percolation of solid‐state sulfur‐based cathode films. In principle, ionic percolation can be increased via the reduction of tortuosity by tailoring electrode microstructure. Furthermore, strategies to reduce the remaining porosity, for example, tailored densification protocols and filling with liquid/polymer electrolytes, can be expected to decrease the tortuosity of cathode film.^[^ ^89^ ^]^ Notably, the inhomogeneous distribution of cathode particles causes high porosity and tortuosity within the composite cathodes, thereby adversely impacting the electrochemical performance of the battery. Recently, an in situ method for the fabrication of composite cathode was applied to ensures the uniform distribution of the components, establishing a low‐tortuosity composite cathode that ensures good electronic and ionic percolation.^[^ ^96^ ^]^

The fabrication pressure plays an important role in affecting the porosity and ion conductivity of solid‐state sulfur cathodes. While the direct evidence on the cathode side is still lacking so far, the studies on SE pellets shed light on the effect of fabrication pressure on the density, grain size and porosity. For example, for the fabrication of the Li_6_PS_5_Cl SE pellets, larger grain size and higher density can be achieved when increasing the external pressure from 50 to 370 MPa. Correspondingly, the denser internal structure of Li_6_PS_5_Cl SE pellets obtained under high pressure of 370 MPa can be obtained with fewer defects and grain boundaries, which may hinder ionic transport. This results in an enhanced room‐temperature ionic conductivity (2.28 mS cm^−1^) in comparison with those under 50 MPa pressure (0.99 mS cm^−1^).^[^ ^97^ ^]^ In addition, the binder content is a processing‐related property, which significantly influence ionic percolation. The high electrochemical performance of a solid‐state cathode should minimize the binder amount and void space, which are two processing‐rooted properties.^[^ ^98^ ^]^

From the battery level, it is recognized that the stacking pressure significantly affects the electronic and ionic percolation of ASSLSBs. In pressure‐lacking batteries, the poor contacts between each component in the cathode greatly hinder the percolation of electrons and ions (Figure 5c). In contrast, the increase of stack pressure enhanced contacts between the electrodes and each component. While the increased stack pressure is beneficial for the electrochemical performance, excessive pressure (higher than 500 MPa) may cause serious problems, including crack generation in the components. In addition, deformation of the current collectors and short‐circuit originating from Li dendrite growth can also be found. More importantly, employment of external pressure greatly affects the materials’ structure and properties. For example, the density of the SE pellets using uniaxial pressing is lower than that using isostatic pressing.^[^ ^99^ ^]^ Therefore, both the stack pressure and the method of applying it need to be carefully investigated during the operation of ASSLSBs to achieve optimal electrochemical performance.

### 5.2 Electrochemical‐Mechanical Coupling Effect

The considerable volume change of sulfur during the charge–discharge processes leads to a significant challenge of electrochemical‐mechanical degradation in thick solid‐state sulfur cathode films.

**Figure 6:**
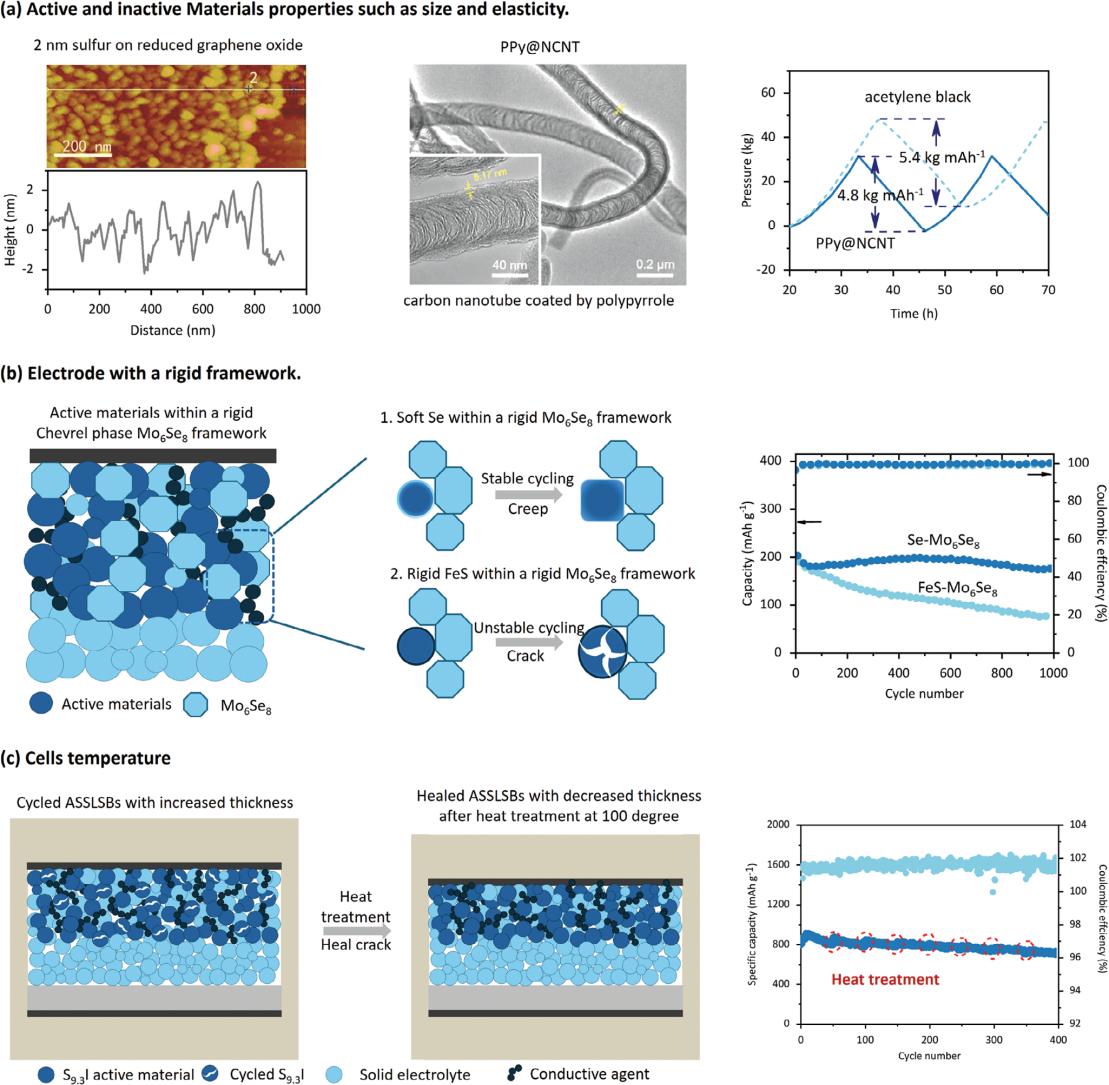
Strategies to overcome electrochemical‐mechanical coupling effect during cycling of the thick sulfur‐based cathode films. a) From the level of the materials, particle size and elasticity of the materials. Reproduced with the permission.^[^ ^21^, ^67^ ^]^ 2017, John Wiley and Sons. 2024, John Wiley and Sons. b) From the level of the electrodes, the induction of creep active materials via rigid framework. Reproduced with the permission.^[^ ^101^ ^]^ 2024, Springer Nature. c) From the level of the cell, external field of heat treatment can fix the crack interface. Reproduced with the permission, 2024, Springer Nature.^[^ ^90^ ^]^

From the material level, a mechanistic study reveals that reducing the particle size in cathode composites by ball milling is effective in buffering volume changes, resulting in a capacity >1000 mAh g^−1^. Therefore, engineering materials structure and size distribution are promising strategies for mitigating chemo‐mechanical failure in ASSLSBs.^[^ ^46^ ^]^ The utilization of robust carbon scaffold may help to reduce the fraction of carbon and maintain the ion conductivity pathways. One example is the introduction of conductive substrate into the cathode composites. This helps to reduce the size of sulfur active species, thus mitigating the volume change over cycling and restricting the electrochemical‐mechanical degradation of solid‐state cathodes. By conformal coating ≈2 nm sulfur onto reduced graphene oxide (**Figure** **6**a), the nanocomposite demonstrates uniform volume changes during cycling, resulting in significantly reduced interface resistance and volume change of the sulfur cathodes. The obtained ASSLSBs operated under 60 °C demonstrates a reversible capacity of 830 mAh g^−1^ under 1675 mA g^−1^.^[^ ^21^ ^]^ Recently, with the direct growth of the SE on FeS_2_ microstructure, the chemo‐mechanical and interfacial issues can be significantly inhibited and satisfying electrochemical performance can be achieved.^[^ ^100^ ^]^

In addition, integrating a continuous and elastic network into solid‐state sulfur electrodes has been reported to regulate mechano‐electrochemical action in ASSLSBs. For example, a nitrogen‐doped carbon nanotube (NCNT) network coated by polypyrrole (PPy) provides a continuous and conductive pathway, reducing the tortuosity of charge transport. The operando pressure measurements tests are conducted, demonstrating that the flexible and viscoelastic structure of PPy@NCNT alleviates the axial pressure on the cathode by 11% with a sulfur areal capacity of 3 mg cm^−2^ at 30 °C (Figure 6a). At a current density of 0.26 mA cm^−2^, the pressure changes for PPy@NCNT and acetylene black are 4.8 and 5.4 kg mAh^−1^, Remarkably, it can achieve an aerial capacity of over 8.8 mAh cm^−2^ and stable cycling under 60 °C.^[^ ^67^ ^]^

From the electrode level, using electrochemically induced stress to drive the active materials deformation can help with formation of stable interfaces. This may fundamentally resolve electrochemical‐mechanical issues. A recent work reports the design of creep‐type cathodes by incorporating a rigid Chevrel phase Mo_6_Se_8_ framework to effectively induce stress and drive creep in Se particles, enhancing interfacial contact and releasing lithiation stress. This 40 wt.% Se + 60 wt.% Mo_6_Se_8_ (Se‐Mo_6_Se_8_) cathode shows lower porosity than conventional cathodes under applied stack pressures from 100 to 360 MPa (Figure 6b). The Se‐Mo_6_Se_8_ cathode maintains good cycling stability with a retention of 81.8% in 1000 cycles. However, the capacity of the FeS‐Mo_6_Se_8_ presents a capacity retention of 35% in 1000 cycles.^[^ ^101^ ^]^

Furthermore, the electrochemical‐mechanical degradation of solid‐state sulfur electrodes can be suppressed by the rational operation of battery cycling. A recent study highlights that conducting a heating‐cooling process on the cycled battery cells can induce melt‐driven interfacial repair and enhance the cycling stability of solid‐state batteries. Specifically, an S_9.3_I cathode with ≈19 µm thickness shows intimate interfacial contact with the LPS layer, while the volume change of the cathode during cycling lead to increased thickness of 22 µm and evident interfacial voids after 50 cycles at 25 °C. After being repeatedly reheated to 100 °C and cooled back to 25 °C, the cycled S_9.3_I cathode with low melting point undergoes in situ interfacial repair, which can be demonstrated in the enhanced cycling stability (Figure 6c). In addition, the degradation of SEs is progressive, leading to poor capacity retention by slowing down lithium‐ion transport and increasing the sulfur‐based cathode overpotential. Furthermore, the electrochemical stability window may be narrow. Potential‐dependent impedance analysis has identified the optimal potential window for cell cycling, which achieves a boosted capacity without significantly deteriorating Li ion transport. Through cycling parameter optimization, a capacity retention of 81.8% at the 100th cycle with an aerial capacity of 3.68 mAh cm^−2^ has been achieved.^[^ ^102^ ^]^

## 6 Conclusion and Outlook

Over the past decade, substantial progress has been achieved in developing ASSLSBs, which represent a promising candidate for next‐generation energy storage technology. The lightweight and high capacity of sulfur make ASSLSBs particularly attractive for applications requiring high gravimetric energy density, such as air mobility energy storage. Additionally, the low operating voltage of sulfur cathode can enhance the safety of ASSLSBs, although this may to some extent limit the power density. Therefore, the ASSLSBs demonstrate both remarkable benefits such as low cost, light weight, high energy density, enhanced safety, and some weaknesses of instability and low power density. Ongoing efforts are required to address existing challenges and obstacles for the development of ASSLSBs.

In terms of the active materials, sulfur remains the most attractive candidate to achieve high energy density of ASSLSBs due to its remarkable theoretical capacity, yet its poor conductivity and significant volume expansion of the cathode film need to be addressed before large‐scale applications. Doping sulfur to form sulfide‐based cathodes provides a promising strategy to address these challenges. However, the introduction of non‐active materials may reduce the specific energy. Li_2_S, with a higher specific energy compared to other transition metal sulfides, however, is very sensitive to the moisture in air, making the storage and cathode preparation difficult on the production line. In addition, the activation barrier of Li_2_S is reported to be decreased by using the catalysts.^[^ ^103^, ^104^, ^105^ ^]^ Recently, SPAN attracts research interest because of its higher stability compared with using elemental sulfur.^[^ ^106^, ^107^ ^]^

For the electrolyte materials in the sulfur‐based cathode films, solid polymer electrolytes (SPEs) exhibit good flexibility, high air‐stability, high chemical/electrochemical stabilities, and good processibility. However, the low ionic conductivity of ≈10^−6^ S cm^−1^ at room temperature and shuttle effect are the main challenges to be considered. Exploring SPEs with high Li^+^ transference numbers and polysulfides‐insoluble SPEs may be promising to address these issues. Sulfide SEs possess the highest ionic conductivities among all reported SSEs. However, the electrochemical instabilities of the sulfide SEs is another important challenge for the practical applications.^[^ ^108^ ^]^ Developing novel SEs such as halide Li_3_YBr_6_ with high ionic conductivities and thermal stability is necessary.^[^ ^109^ ^]^ In addition, the employment of polymer coatings or polymer‐inorganic composite in which the cohesive force provided by glutinous layers can effectively address structure detachment and solid‐solid contact loss.^[^ ^110^ ^]^ This offers a promising solution for manufacturing of the sulfur‐based cathode films with high ionic conductivity and adhesive strength.^[^ ^111^, ^112^, ^113^ ^]^

Preparation of large‐size, sheet‐type thick sulfur‐based cathode film is crucial for industrial scalability of ASSLSBs. In SPEs systems, it is straightforward to adapt the traditional slurry casting methods for ASSLSBs by replacing the binder with SPEs to facilitate Li^+^ percolation. Extrusion is an effective method for producing large‐size SPE‐based electrodes. With sulfide electrolytes, slurry casting remains a viable technology. Future efforts could be made to develop new binders that offer improved viscoelasticity and solubility in non‐polar solvents. Additionally, developing novel electrolytes with good stability against polar solvents via doping soft acid elements and substituting soft alkali sulfur with hard alkali oxygen could be effective.^[^ ^114^ ^]^ However, considering potential side reactions between current SEs and solvents, the dry film method seems more promising, as it preserves the properties of binders and SEs.^[^ ^115^ ^]^ Additionally, the binder content can be reduced to as low as 0.1 wt %, significantly minimizing the adverse effects of the binder on lithium transport. Other processes such as liquid‐phase synthesis and electrospinning can be conducted under ambient conditions, with high production efficiency possesses scalable potential.^[^ ^116^ ^]^ For commercialization of ASSLSBs, a diversified process strategy should be adopted for various materials and systems. Especially under conditions of increased sulfur loading, extended cycle life, and high current density, a rational combination of different processes can balance and compensate for the shortcomings of a single process.

The cell failure caused by electrochemical‐mechanical coupling effect in the sulfur cathode film is a significant obstacle to the ASSLSBs. Wang et al. demonstrated that all‐solid‐state batteries with glass and glass‐ceramic SE mitigate contact loss between the components compared with using crystalline SE.^[^ ^117^ ^]^ Therefore, the mechanical properties (such as Young's modulus) of each component including active materials and inactive components need to be considered for suppressing electrochemical‐mechanical degradation for the ASSLSBs.^[^ ^118^ ^]^ For example, adopting flexible, pressure self‐adaptable binders can effectively alleviate volume change of cathodes during cycling. Additionally, integrating electrochemical and mechanical properties of active and inactive materials can be effective. For instance, introducing a rigid framework to induce creep of the active materials. The change of external field of the cell is also a potential solution, techniques such as isostatic pressing and external heating have recently been developed to mitigate the electrochemical‐mechanical degradation, which has demonstrated a positive effect on enhancing the cycling stability and capacity output of coin cells and has the potential to be shifted to pouch cells. In addition, introducing void spaces in the electrode to elevate the volume change may not be an effective solution for the solid‐state sulfur‐based cathodes, as it can reduce ion permeability and lower the energy density.

Finally, the mechanism underlying sulfur solid‐state redox processes remains unclear. In situ characterization techniques, such as focused ion beam‐scanning electron microscopy analysis, X‐ray computed tomography, operando visualization and multi‐dimensional microscopic stress mapping with real‐time capabilities offer more informative insights compared to ex situ methods.^[^ ^111^ ^]^ Furthermore, theoretical methods that involves testing conditions, including temperature, pressure, and voltage, should be applied to achieve profound insights.

## Conflict of Interest

The authors declare no conflict of interest.
